# Metabolomic and microbial insights: Kai-Xin-San’s impact on Alzheimer’s disease pathology

**DOI:** 10.1016/j.isci.2025.112817

**Published:** 2025-06-03

**Authors:** Huifen Ma, Zhiyang Yu, Qiong Qiao, Wenpan Wang, Zhonghua Li, Pan Wang, Junying Song, Xiaowei Zhang, Yunfang Su, Yiran Sun, Zhishen Xie, Zhenqiang Zhang

**Affiliations:** 1 Collaborative Innovation Center of Prevention and Treatment of Major Diseases by Chinese and Western Medicine, Henan University of Chinese Medicine, Zhengzhou, Henan 450046, China; 2 Collaborative Innovation Center of Research and Development on the Whole Industry Chain of Yu-Yao, Zhengzhou, Henan 450046, China; 3 Academy of Chinese Medical Sciences, Henan University of Chinese Medicine, Zhengzhou, Henan 450046, China; 4 School of Pharmacy, Chengdu Medical College, Chengdu 610500, China

**Keywords:** Molecular neuroscience, Metabolomics

## Abstract

There has been increasing interest in the connection between AD, gut microbiota, and metabolites. Kai-Xin-San (KXS) has been commonly employed in ancient and modern Chinese clinical trials for the treatment of dementia; however, whether the protective effect of KXS in AD is related to the gut microbiota remains elusive. APP/PS1 mice were used as the model of AD. 43 key metabolites influenced by KXS were screened using untargeted metabolomics. At the genus level, Clostridium_IV, Eubacterium, Acetatifactor, etc., were identified to be impacted by KXS using 16S rRNA sequencing. Additionally, we identified 9 distinct intestinal floras at the genus level that were correlated with 13 pivotal differential metabolites related to cognitive impairment. KXS also inhibited the neuroinflammation, mostly via regulating the key metabolites. A potential relationship between gut microbiota, metabolites, and neuroinflammation is suggested as a protective mechanism of KXS in AD. These findings provide support for further development of KXS.

## Highlights

• KXS can exert the anti-AD effect by regulating the composition of the gut microflora
• KXS modulates gut microbiota composition and further influences metabolites
• The inhibition of neuroinflammation by KXS is related to the regulation of metabolites

## Graphical abstract

**Figure:**
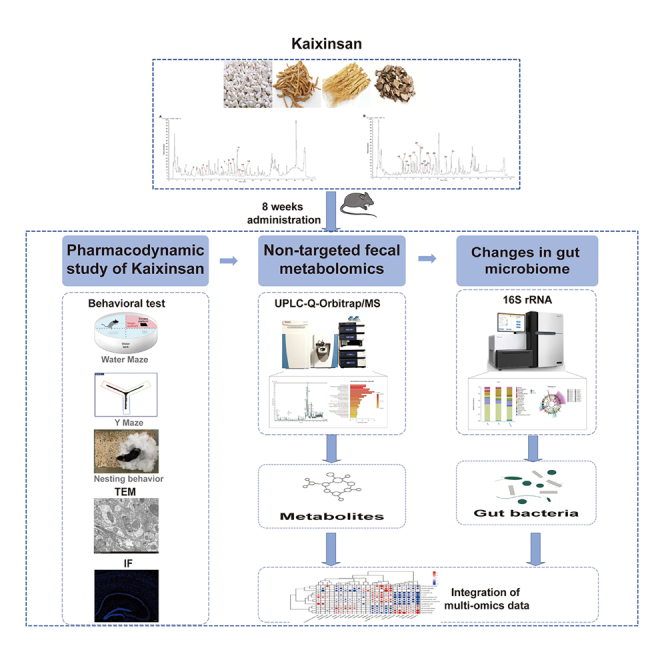

## Introduction

The prevalence of Alzheimer’s disease (AD), a disease of aging-related neurodegeneration, is increasing due to the aging population worldwide.^1^ Based on the World Alzheimer Report for 2019, AD is one of the leading causes of elderly morbidity and mortality.^1^ Clinically, patients with AD are diagnosed based on classical symptoms and cognitive profiles, including progressive memory loss, cognitive and personality dysfunction, behavioral changes, and even basic losses in bodily function.^2^ Pathologically, AD is considered a multifactorial disease closely related to multiple damaging factors, such as extracellular beta amyloid (Aβ) deposition, intracellular tau protein accumulation, neuroinflammation, and neuronal loss.^2^

A significant study published in the esteemed journal *Science* revealed interconnections between the microbiota, neuroinflammation, and tau-mediated neurodegeneration, highlighting the effect of the gut microbiota in the progression of AD through a direct influence on tau pathology.^3^ Currently, most treatments provide only limited relief from symptoms and fail to halt or reverse the progressive deterioration of AD.^2^ Therefore, AD remains an irreversible and incurable neurodegenerative disorder, necessitating the need for the urgent discovery of innovative therapeutic agents with novel mechanisms of action.

Traditional Chinese medicine (TCM) has been extensively employed in China to treat complex diseases for an extended period due to its favorable characteristics, including multiple components, efficacies, and targets.^4^ Several TCM formulations have exhibited potent neuroprotective effects in multiple models of AD.^5^^,^^6^^,^^7^ Kai-Xin-San (KXS) is first found in the book of Bei Ji Qian Jin Yao Fang written by Sun Simiao during the Tang dynasty, which consists of *Ginseng Radix*, *Polygalae Radix*, *Acori Tatarinowii Rhizoma*, and *Poria*. As a classic formula, KXS has been commonly employed in ancient and modern Chinese clinical trials for the treatment of dementia. According to the previous reports, KXS can ameliorate cognitive dysfunction in an animal model of AD by promoting Aβ_42_ degradation, inhibiting neuroinflammation, and increasing cholinergic and glutamatergic neurotransmission.^8^^,^^9^^,^^10^ More importantly, KXS has been reported to regulate gut microbiota in mice with chronic unpredictable mild stress-induced depression.^11^ However, the specific pharmacological mechanisms by which KXS regulates gut microbiota and exerts its effects against AD remain challenging to elucidate. Accordingly, this work aimed to reveal the underlying mechanism of KXS using a model of AD based on amyloid precursor protein/presenilin 1 (APP/PS1) mice.

## Results

### Active chemical components in KXS

**Figure 1:**
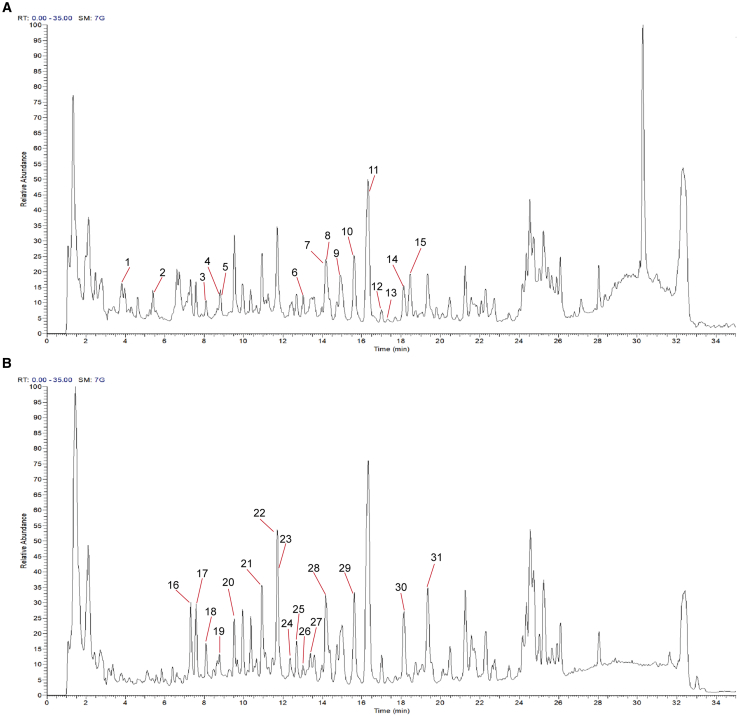
The total ion chromatogram of KXS (A and B) Positive ion mode (A) and Negative ion mode (B). Representative components were determined as follows: 1. Senegin-II, 2. Notoginsenoside R1, 3. Onjisaponin B, 4. Notoginsenoside A, 5. Ginsenoside Rc, 6. Quinquenoside R1, 7. Ginsenoside Rb1, 8. Ginsenoside Rg1, 9. Notoginsenoside R2, 10. Ginsenoside Rs1, 11. Ginsenoside Rs2, 12. Onjisaponin F, 13. Ginsenoside Rg2, 14. Pseudoginsenoside F11, 15. Ginsenoside Ro, 16. Sibiricose A5, 17. Sibiricose A1, 18. Dehydrotumulosic acid, 19. Poricoic acid AE, 20. Polyporenic acid C, 21. Sibiricose A3, 22. Sibiricaxanthone B, 23. Sibiricaxanthone A, 24. Poricoic acid A, 25. Tenuifoliside B, 26. Polygalaxanthone II, 27. 3,6-Disinapoylsucrose, 28. Dehydropachymic acid, 29. Tenuifoliside A, 30. Pachymic acid, 31. Glomeratose A.

The analysis of KXS sample was performed on the UHPLC-OE/MS for determining the chemical components. The total ion chromatogram of KXS was showed as the positive and negative modes indicating the chemical composition (Figures 1A and 1B). Referring to the Integrative Pharmacology-based Research Platform of TCM^12^ and publications on the compositions of KXS and the four Chinese medicinal herbs, the potential compounds of KXS were detected and subsequently identified 31 representative ingredients.

### Effects of KXS on learning ability and memory impairment in APP/PS1 mice

**Figure 2:**
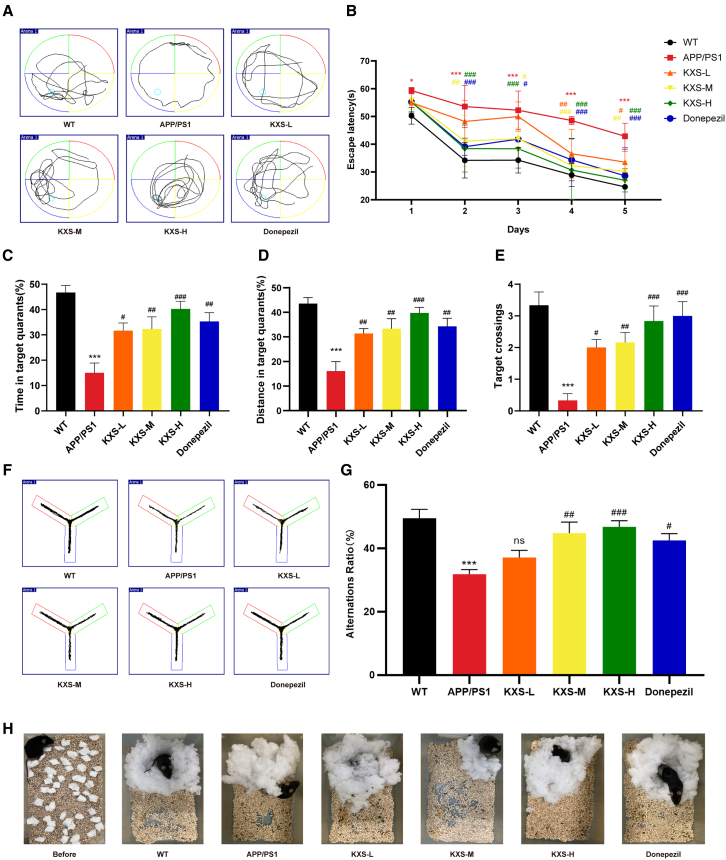
Effects of KXS on learning and memory impairment of APP/PS1 mice (A) The representative motion trajectory in the water maze test (*n* = 8). (B) The escape latency in positioning navigation training. (C) The percentage of time that mice spend in the target quadrant in the space exploration experiment. (D) The distance percentage of the mice in the target quadrant. (E) The number of times the mice crossed the platform. (F) The representative motion trajectory in the Y-maze test (*n* = 8). (G) Spontaneous alternation. (H) Representative images in the nesting behavior test. ^∗^*p* < 0.05, ^∗∗^*p* < 0.01 and ^∗∗∗^*p* < 0.001, compared with the WT group; ^#^*p* < 0.05, ^##^*p* < 0.01 and ^###^*p* < 0.001, compared with the APP/PS1 group, ns: no significance.

To assess the learning and memory abilities of APP/PS1 mice and the potential therapeutic effects of KXS, the MWM, Y-maze, and Nestbuilding test were employed. The results showed that APP/PS1 mice took longer time to reach the platform than WT mice. However, time was reduced in the KXS group in a dose-dependent manner, with an effect equivalent to that of the mice in the donepezil group (Figures 2A and 2B). After 5 days of training, the spatial memory ability of the mice was evaluated using a space exploration experiment on day 6. Compared with WT mice, APP/PS1 mice spent a lower percentage of time in and distance near the target quadrant and crossed the platform fewer times. These indexes were significantly improved by KXS and donepezil (Figures 2C–2E). Figures 2F and 2G showed that the ratio of spontaneous alternation was decreased in APP/PS1 mice and markedly increased after treating with KXS. The Nestbuilding test exhibited that the nests made by KXS-treated mice were obviously stronger than APP/PS1 mice. Therefore, these findings indicate that KXS effectively restored cognitive dysfunction in APP/PS1 mice.

### Effects of KXS on synapses in the hippocampus of APP/PS1 mice

**Figure 3:**
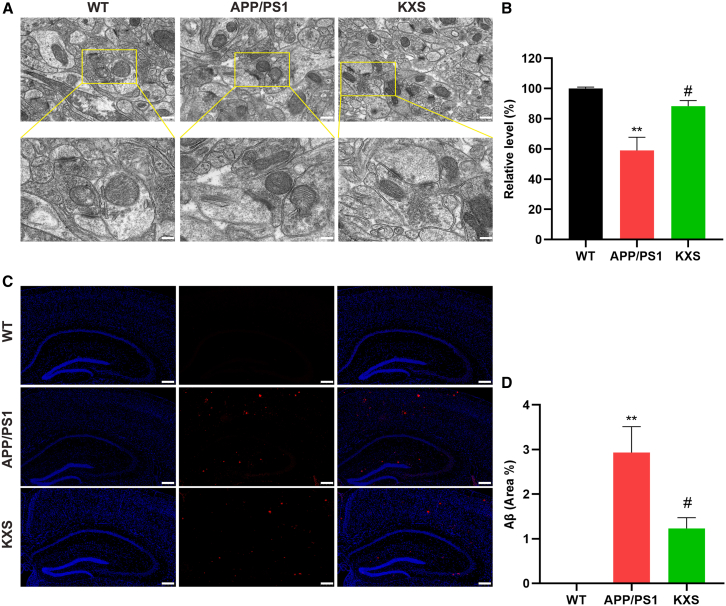
Effects of KXS on synapses and Aβ burden in APP/PS1 mice (A) Representative images of the synapses of hippocampal neurons in mice (scale = 1 μm, 0.5 μm). (B) Level of synapses in the different groups (*n* = 3). (C) Representative images of immunofluorescence staining of Aβ in the brains (scale = 200 μm). (D) Quantitative analysis of Aβ in the different groups (*n* = 3). ^∗∗^*p* < 0.01, compared with the WT group; ^#^*p* < 0.05, compared with the APP/PS1 group.

To further evaluate the potential role of KXS in the treatment of AD, electron microscopy and immunofluorescence staining were used to study the histopathological changes in the brain of the different groups. It is known that the neuronal synapses are closely related to cognitive performance.^2^ As shown in Figure 3A, the number of synapses was reduced, and the ultrastructure of the synapses was obviously degraded in the hippocampus of APP/PS1 mice compared with WT mice. Upon intervention with KXS, the synaptic structure of the neurons was significantly improved compared to the APP/PS1 group. Immunofluorescence staining revealed that neurons in the WT group were tightly arranged with no significant Aβ deposition, unlike those in the APP/PS1 group (Figure 3B). Aβ deposition was increased in the neurons of the APP/PS1 group, which was significantly reduced by treatment with KXS. The results reveal that KXS can improve synaptic dysfunction and inhibit the deposition of Aβ in AD mice.

### Effects of KXS on the fecal metabolites of APP/PS1 mice

**Figure 4:**
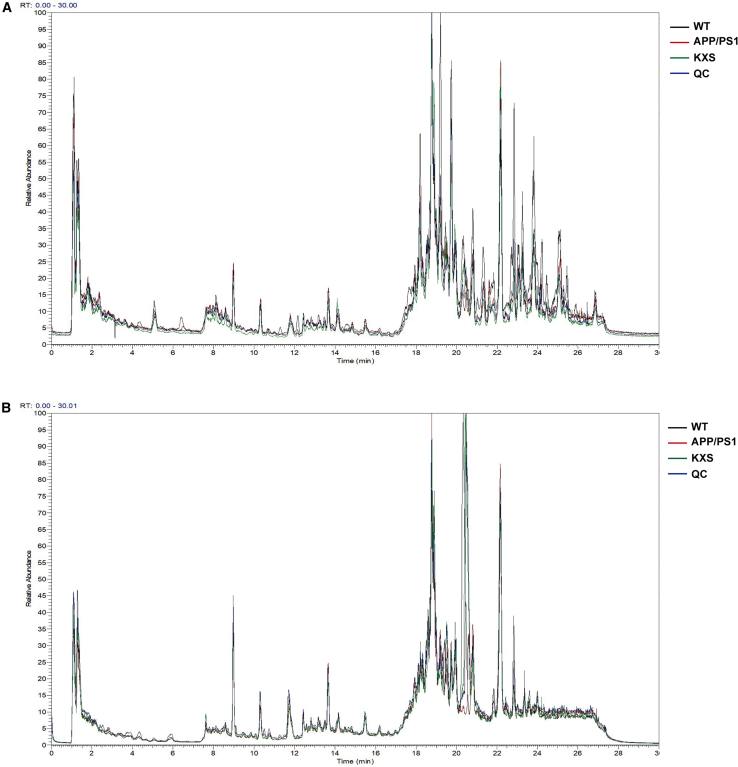
Fecal metabolic profiling of each group (A and B) Fecal metabolic profiling of each group in positive ion mode (A) and negative (B) ion mode.

**Figure 5:**
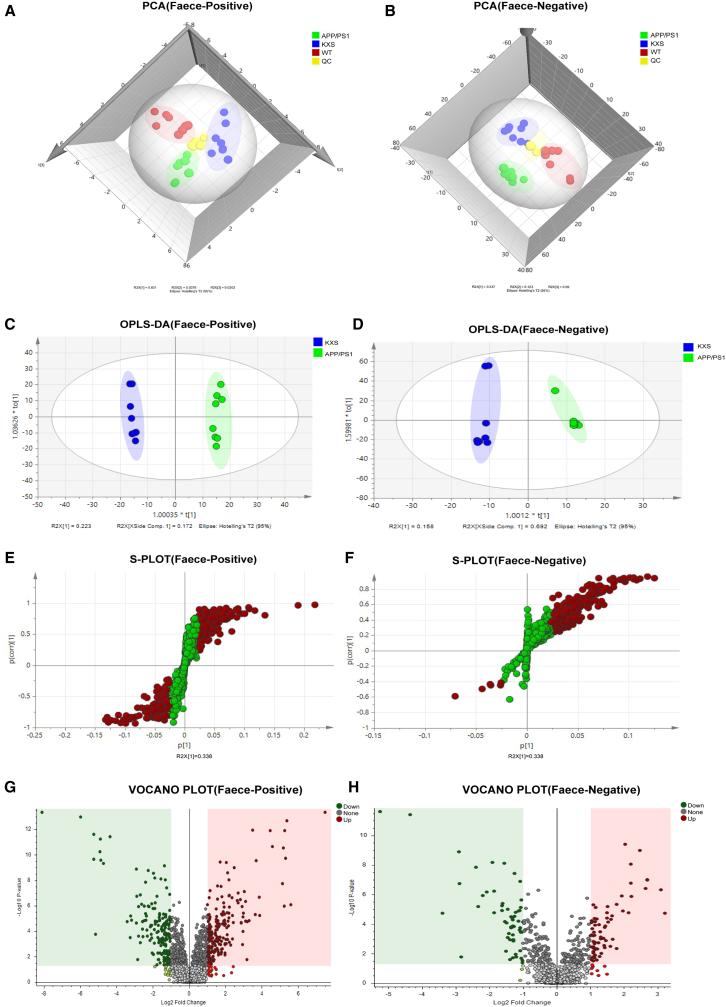
Metabolite analysis between different groups (A) PCA analysis of different groups in positive ion mode. (B) PCA analysis of different groups in negative ion mode. (C) OPLS-DA analysis of APP/PS1 and KXS group in positive ion mode. (D) OPLS-DA analysis of APP/PS1 and KXS group in negative ion mode. (E) S-plot diagram for APP/PS1 and KXS group in positive mode. (F) S-plot diagram for APP/PS1 and KXS group in negative mode. (G) Volcanic diagram of fecal metabolites of APP/PS1 and KXS mice in positive mode. (H) Volcanic diagram of fecal metabolites of APP/PS1 and KXS mice in negative mode.

To explore the mechanism of neuroprotection exerted by KXS, a non-targeted metabolomics analysis of feces was conducted. Typical positive and negative total ion chromatograms (TIC) of representative fecal samples from the WT, APP/PS1, and KXS groups are shown in Figures 4A and 4B. PCA analysis was performed on the data of the WT, APP/PS1, KXS, and QC groups (Figures 5A and 5B). The results showed that the sample data in the QC group was tightly clustered, which further indicated that the method was relatively stable. Under the positive and negative ion modes, the data of WT, APP/PS1, and KXS group were obviously separated, which indicated that altered endogenous metabolites in the fecal matter of APP/PS1 mice were regulated by KXS. Additionally, the position of the KXS group was closer to the WT group, indicating that the metabolite level of APP/PS1 mice tended to be normal following KXS treatment.

OPLS-DA analysis revealed that APP/PS1 group and KXS group showed a clear separation in positive and negative ion mode, which indicated that there were significant differences between the two groups (Figures 5C and 5D). In the corresponding S-plot generated from OPLS-DA, the variables farther away from the origin were considered as more significant, which are the key ions to distinguish the KXS group from the AD model group (Figures 5E and 5F). Therefore, these variables can be regarded as potential biomarkers. The volcanic plot showed that there were 301 differential ions between the KXS group and APP/PS1 group, of which 147 were upregulated and 154 were downregulated (Figures 5G and 5H). According to the VIP value > 1, the ions were further screened, and the differential ions were characterized by the database. A total of 43 key differential metabolites were screened out, which were significantly changed in APP/PS1 mice and recalled by KXS, including echinacoside, 4-methyl-5-hydroxyethyl thiazole, 2-*O*-ethyl ascorbic acid, propionyl carnitine, and nitrotyrosine (Table 1).

**Table 1:** Identified differential metabolites

Ionization mode	Name	Formula	m/z	RT [min]	Reference Ion	VIP	AD/Normal	KXS/AD	Pathways
ESI+	Glutamic acid	C5 H9 N O4	148.06072	1.254	[M + H]+1	1.63226	−1.39↓^c^	0.77↑^b^	Histidine metabolism
	L-Glutamine	C5 H10 N2 O3	147.07673	1.249	[M + H-NH3]+1	1.43044	−0.53↓^a^	0.7↑^b^	Glutamate Metabolism, Urea Cycle
	Nicotinamide	C6 H6 N2O	123.05549	1.851	[M + H]+1	1.67072	−0.61↓^c^	1.84↑^b^	Nicotinate and nicotinamide metabolism
	PA(21:0/0:0)	C24 H49O7 P	481.32963	20.686	[M + H]+1	1.5406	−0.59↓^a^	0.97↑^b^	Triacylglycerol Biosynthesis
	Tryptophan	C11 H12 N2 O2	188.07095	8.124	[M + H]+1	1.96716	−4.77↓^c^	2.21↑^c^	Tryptophan metabolism
	Estrone	C18 H22 O2	271.1659	8.416	[M + H]+1	1.50073	−3.07↓^c^	1.13↑^b^	Estrogen Metabolism
	4-Pyridoxic acid	C8 H9 N O4	184.06068	2.285	[M + H]+1	1.56083	−0.76↓^c^	0.41↑^c^	Vitamin B6 Metabolism
	Urocanic acid	C6 H6 N2 O2	139.05053	1.372	[M + H]+1	1.8898	−3.06↓^c^	2.7↑^c^	Histidine metabolism
	N-Acetylneuraminic acid	C11 H19 N O9	310.11382	1.298	[M+Na]+1	1.8427	−0.92↓^a^	2.23↑^c^	Amino Sugar Metabolism
	Xanthurenic acid	C10 H7 N O4	206.045	8.691	[M + H]+1	1.66009	−4.61↓^c^	1.13↑^c^	Tryptophan metabolism
	Estriol	C18 H24 O3	311.16093	8.54	[M + H]+1	1.99096	−0.93↓^c^	2.48↑^c^	Estrone Metabolism
	Leucine	C6 H13 N O2	132.10212	1.299	[M + H]+1	1.59917	−2.15↓^c^	1.45↑^c^	Valine, leucine and isoleucine degradation
	Succinic anhydride	C4 H4 O3	101.02341	2.529	[M + H]+1	1.65413	−1.07↓^c^	1.12↑^c^	–
	Docosahexaenoic acid	C22 H32 O2	329.24799	25.12	[M + H]+1	1.47217	−1.28↓^c^	1.14↑^b^	alpha-Linolenic acid metabolism
	4-Imidazolone-5-propanoate	C6 H8 N2 O3	335.09554	1.316	[M+Na]+1	1.75754	−3.15↓^c^	1.96↑^c^	Histidine metabolism
	Urothion	C11 H11 N5 O3 S2	326.03846	8.394	[M+ACN+H]+1	1.8137	−0.6↓^c^	0.91↑^c^	–
	2,4,6-triaminotoluene	C7 H11 N3	138.10281	1.123	[M + H]+1	1.46764	−0.82↓^c^	0.94↑^c^	–
	Methyl 2,3-dihydro-3-hydroxy-2-oxo-1H-indole-3-acetate	C11 H11 N O4	244.05847	11.585	[M+Na]+1	1.44953	−0.59↓^b^	0.51↑^b^	–
	Thermozeaxanthin-15	C61 H94 O8	955.70141	21.318	[M + H]+1	1.36348	−1.25↓^c^	0.43↑^b^	–
	5-Methoxyindoleacetate	C11 H11 N O3	228.06366	13.009	[M + H]+1	1.70592	−1.45↓^c^	0.69↑^c^	Tryptophan metabolism
	leu-gln	C11 H21 N3 O4	260.1612	7.493	[M + H]+1	1.70212	−0.39↓^b^	0.45↑^b^	–
	7alpha,12alpha-Dihydroxy-3-oxochol-4-en-24-oic Acid	C24 H36 O5	405.26422	19.604	[M + H-NH3]+1	1.29788	−0.94↓^c^	0.72↑^b^	–
	Styrene	C8 H8	105.06995	6.466	[M + H]+1	2.06842	1.92↑^c^	−5.26↓^c^	–
	N-{3-[(4-Acetamidobutyl)amino]propyl}acetamide	C11 H23 N3 O2	230.18668	2.005	[M + H]+1	1.63236	0.6↑^a^	−1.03↓^c^	–
	5-Hydroxyindole	C8 H7 N O	134.06026	10.993	[M + H]+1	1.80286	1.68↑^c^	−1.06↓^c^	Tryptophan metabolism
	N8-Acetylspermidine	C9 H21 N3 O	188.17613	1.198	[M + H]+1	1.65254	0.74↑^b^	−0.97↓^c^	–
	(11E,15Z)-9,10,13-trihydroxyoctadeca-11,15-dienoic acid	C18 H32 O5	346.25964	17.374	[M + H]+1	1.61792	1.06↑^c^	−0.97↓^c^	–
	D-ribosylnicotinate	C11 H13 N O6	256.08212	1.353	[M + H]+1	1.56376	−1.27↓^b^	1.88↑^c^	Nicotinate and nicotinamide metabolism
	N-oleoyl glycine	C20 H37 N O3	381.31175	17.635	[M+Na]+1	1.71974	1.05↑^c^	−0.62↓^c^	Fatty Acids metabolism
	3-Methylindole acetate	C11 H11 N O2	212.06853	14.266	[M + H]+1	1.72703	0.89↑^c^	−1.1↓^c^	Tryptophan metabolism
	L-Tyrosine methyl ester	C10 H13 N O3	196.0974	7.587	[M + H]+1	1.8989	0.48↑^c^	−0.54↓^c^	–
	O-Propanoylcarnitine	C10 H19 N O4	218.13912	8.726	[M + H]+1	1.74212	0.55↑^a^	−0.97↓^c^	Oxidation of Branched Chain Fatty Acids
	L-3-nitrotyrosine	C9 H10 N2 O5	227.06617	14.848	[M + H]+1	1.73455	0.79↑^c^	−1↓^c^	–
	Testolate	C19 H28 O4	321.20414	21.946	[M+Na]+1	1.75596	1.43↑^c^	−0.86↓^c^	–
	1-(alpha-D-Glucopyranosyluronosyl)-3-[(2S)-1-methyl-5-oxo-2-pyrrolidinyl]pyridinium	C16 H20 N2 O7	353.1351	10.618	[M + H]+1	1.46177	1.49↑^c^	−0.9↓^b^	Nicotine Metabolism Pathway
	N-(2E,9Z-hexadecadienoyl)-homoserine lactone	C20 H33 N O3	336.25412	13.414	[M + H]+1	1.35236	0.4↑^c^	−0.27↓^b^	–
	2-*O*-ETHYL ASCORBIC ACID	C8 H12 O6	205.07119	14.85	[M + H]+1	1.77666	0.59↑^b^	−0.97↓^c^	–
	Butyryl-L-carnitine	C11 H21 N O4	232.15475	10.981	[M + H]+1	1.53646	0.97↑^c^	−0.8↓^b^	Fatty Acids metabolism
	2,5,6-Trihydroxy-5,6-dihydroquinoline	C9 H9 N O3	180.06582	8.515	[M+Na]+1	1.53841	0.49↑^a^	−0.84↓^c^	–
	Capryloylglycine	C10 H19 N O3	202.14401	14.254	[M + H]+1	1.84758	2.11↑^c^	−2.2↓^c^	Fatty Acids metabolism
	N-pentylpantothenamide	C14 H28 N2 O4	289.21295	8.443	[M + H]+1	1.17429	0.56↑^b^	−0.41↓^a^	–
ESI-	PE(17:1(9Z)/0:0)	C22 H44 N O7 P	464.27904	23.261	[M-H]-1	2.31764	1.89↑^a^	3.81↑^c^	Phosphatidylcholine Biosynthesis
	4-Hydroxybenzoic acid	C7 H6 O3	137.02482	8.567	[M-H]-1	1.79005	−1.55↓^a^	1.16↑^a^	Ubiquinone Biosynthesis

a *p* < 0.05.

b *p <* 0.01. ↑increase, ↓decrease.

c *p* < 0.001.

### Analysis of metabolic pathways in the non-targeted metabolome

**Figure 6:**
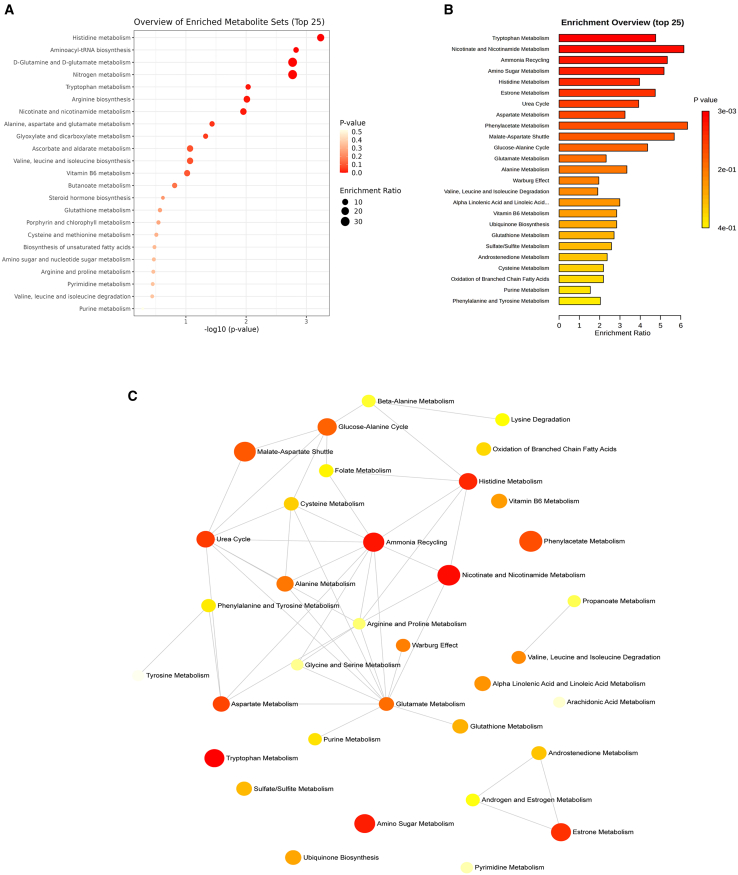
Enrichment analysis of differential metabolites in feces (A) Enrichment bubble diagram of metabolic pathway. (B) Functional enrichment bar chart. (C) The correlation between metabolic pathways.

To determine the metabolic pathways related to KXS regulation, Kyoto Encyclopedia of Genes and Genomes (KEGG) and metabolic pathway analyses were performed with MetaboAnalyst software based on the different metabolites mentioned above. As shown in the Figures 6A and 6B, the significant changes between the KXS and APP/PS1 group mainly involved tryptophan metabolism, histidine metabolism, nicotinic acid and nicotinamide metabolism, ammonia recycling, arginine biosynthesis, amino sugar metabolism, and so on. The correlation between metabolic pathways was analyzed, and the results showed that histidine metabolism, nicotinic acid and nicotinamide metabolism, ammonia recycling, and the urea cycle were the key pathways (Figure 6C).

### Effects of KXS on the composition of the gut microbiota in APP/PS1 mice

**Figure 7:**
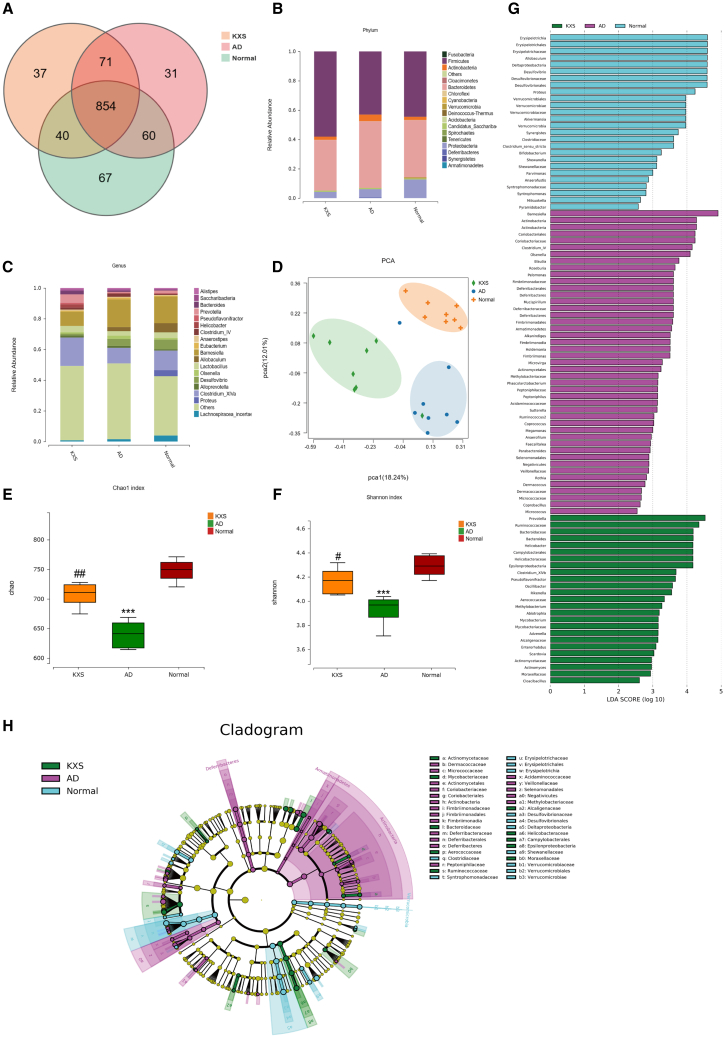
Regulation of gut microbiota in APP/PS1 mice by KXS (A) Number of OTUs common or unique to different groups. (B) Bacterial taxonomic profiling at the phylum levels. (C) Bacterial taxonomic profiling at the genus levels. (D) PCA analysis for all groups at the OTU level. (E) α-diversity analyzed by Chao1 indexes. (F) α-diversity analyzed by Shannon indexes. (G) Derived from LEfSe analysis, showing the biomarker taxa LDA score of >2. (H) Taxonomy cladogram from LEfSe analysis. ^∗∗∗^*p* < 0.001, compared with the normal group; ^#^*p* < 0.05 and ^##^*p* < 0.01, compared with the AD group.

There is accumulating evidence to suggest that the gut microbiota plays an important role in the development of AD.^13^^,^^14^ Furthermore, the metabolites in feces are mainly influenced by the gut microbiota. To explore the role of the gut microflora in the effects of AD treatment with the KXS formula, 16S rRNA sequencing was performed on fecal samples. A total of 989 OTUs were identified in the abovementioned three groups, including 921 OTUs in the WT group, 885 OTUs in the AD group, and 891 OTUs in the KXS group. Among these, 60 OTUs were identified in both the WT and AD groups, and 40 OTUs in both the WT and KXS groups (Figure 7A). Subsequently, the gut microbiota structures of the three groups were evaluated at different levels. At the phylum level, Firmicutes, Actinobacteria, Bacteroidetes and Proteobacteria were the dominant bacteria. In the AD group, the abundance of Firmicutes was lower, while Actinobacteria and Bacteroidetes were higher, compared with the WT group; however, after intervention wit KXS, they were all closer to the WT group (Figure 7B). At the genus level (Figure 7C), the dominant bacterium *Clostridium_XIVa* was obviously lower in the AD group than in the WT group, while it was increased following KXS intervention. The abundance of the dominant bacteria *Barnesiella* in the AD group was higher compared with WT group, while it was significantly reduced after KXS treatment. The PCA analysis, an indicator of β-diversity, showed clear separations among the WT, AD, and KXS groups, exhibiting evidently different microbiota compositions among the three groups (Figure 7D). The parameters of the α-diversity indexes, including Chao1 and the Shannon index, were all obviously lower in APP/PS1 mice than in WT mice, suggesting a significant alteration in α-diversity of the gut microbiota. However, the microbial abundance was significantly increased in the KXS groups (Figures 7E and 7F). In order to investigate the bacteria with higher abundance in the three groups, which have the potential to serve as biomarkers, a LEfSe analysis was performed. Overall, 96 genera were determined with LDA scores >2 (Figure 7G). A cladogram of the annotated branches of different bacteria is shown in Figure 7H. According to these findings, KXS regulates gut microflora composition and has beneficial effects on the host.

### Integrated analysis of the microbiome and metabolomics

**Figure 8:**
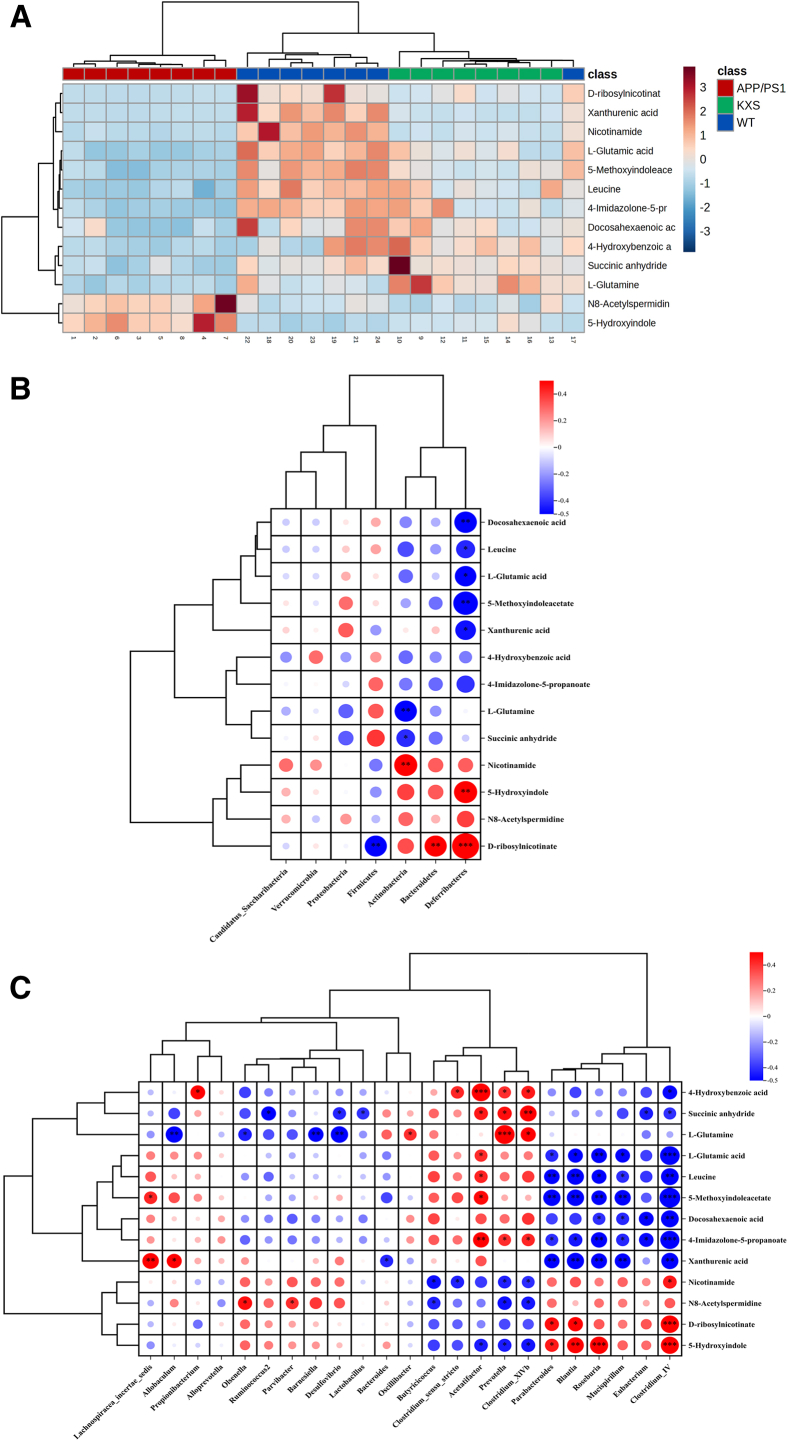
Integrated analysis of microbiome and metabolomics (A) Heatmap analysis of key fecal metabolites, red represents increase, and blue indicates decrease. (B) Correlation heatmap to represent the correlation values between disturbed gut microbial phylum and altered fecal metabolites. (C) Correlation heatmap to represent the correlation values between disturbed gut microbial genera and altered fecal metabolites. Red represents positive correlation, and blue indicates negative correlation. ^#^*p* < 0.05, ^##^*p* < 0.01 and ^###^*p* < 0.001 compared with Kkay by Student’s t test.

According to the enrichment analysis, 13 key metabolites were screened relating to tryptophan metabolism, nicotinic acid and nicotinamide metabolism, histidine metabolism, the urea cycle, and purine metabolism. The trends of the 13 metabolites in each group are shown as a heatmap (Figure 8A). The levels of N8-acetylspermidine, D-ribosylnicotinate, 5-hydroxyindole, and nicotinamide were increased and xanthurenic acid, leucine, succinic anhydride, 4-imidazolone-5-propanoate, 5-methoxyindoleacetate, 4-hydroxybenzoic acid, docosahexaenoic acid, L-glutamic acid, and L-glutamine were decreased in the AD group, while they were significantly reversed following KXS treatment. To investigate whether gut microbiota and metabolites have a functional relationship, spearman correlation analysis was performed. As shown in Figure 8B, the metabolites were mainly related to Deferribacteres, Bacteroidetes, Actinobacteria, and Firmicutes at the phylum level. Based on the sequencing results, the genus-level bacteria were screened, and correlation analyses were performed (Figure 8C). Nine bacteria showed a closely correlation with the key metabolites. 4-hydroxybenzoic acid, succinic anhydride, L-glutamic acid, L-glutamine, leucine, 5-methoxyindoleacetate, docosahexaenoic acid, 4-imidazolone-5-propanoate, and xanthurenic acid were negatively correlated with *Clostridium_IV*, *Eubacterium*, *Mucispirillum*, *Roseburia*, *Blautia*, *Parabacteroides*, and positively correlated with *Acetatifactor*, *Prevotella*, *Clostridium_XlVb*. Furthermore, nicotinamide, N8-acetylspermidine, D-ribosylnicotinate, and 5-hydroxyindole had a positive correlation with *Clostridium_IV*, *Mucispirillum*, *Roseburia*, *Blautia*, and *Parabacteroides*, and a negative correlation with *Acetatifactor*, *Prevotella*, *Clostridium_XlVb*, and *Butyricicoccus*.

### Effect of KXS on metabolism pathway and inflammation

**Figure 9:**
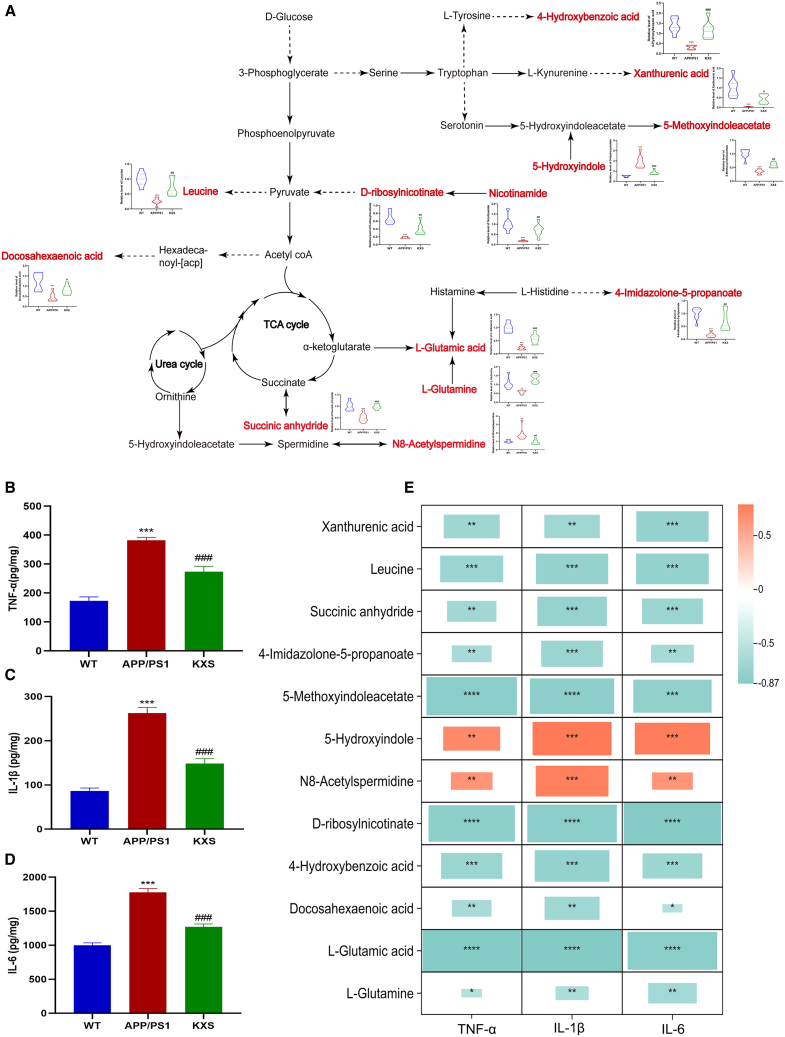
Verification of experimental results (A) The network and trend of major metabolites, (WT, APP/PS1, and KXS are separately the blue, red, and green). (B) The levels of TNF-α in brain. (C) The levels of IL-1β in brain. (D) The levels of IL-6 in brain. (E) Correlation heatmap to represent the correlation values between inflammatory factors and altered fecal metabolites. Data are mean ± SEM. ∗*p* < 0.05, ∗∗*p* < 0.01, ∗∗∗*p* < 0.001 vs. WT group, ^#^*p* < 0.05, ^##^*p* < 0.01, ^###^*p* < 0.001 vs. APP/PS1 group, one-way analysis of variance, Sidak multiple comparison trial.

In the final part of this work, the metabolic network regulated by KXS was constructed based on the determined trend of metabolites. As shown in Figure 9A, the tryptophan metabolites, including xanthurenic acid, 4-hydroxybenzoic acid, and 5-methoxyindoleacetate, strikingly decreased in APP/PS1 mice and increased after treating with KXS. L-glutamic acid, L-glutamine, docosahexaenoic acid exhibited similar trend. The opposite trend was observed in 5-hydroxyindole and N8-acetylspermidine. Additionally, KXS remarkably decreased the levels of TNF-α, IL-1β and IL-6, which increased in APP/PS1 mice (Figures 9B–9D). Furthermore, the Figure 9E indicated that there was a significant positive correlation between 5-hydroxyindole, N8-acetylspermidine and inflammatory factors, while there was a significant negative correlation between other metabolites and inflammatory factors. Leucine, 5-methoxyindoleacetate, D-ribosylnicotinate, and L-glutamic acid possessed the most significant correlation with inflammatory factors. These results suggest that KXS influenced the fecal metabolites and further inhibited inflammation.

## Discussion

AD is a severe neurodegenerative disease characterized by progressive cognitive decline. Recent studies have highlighted the role of an imbalance in the gut microbiota in the development of AD.^13^^,^^14^ Unfortunately, current pharmacological approaches for AD have not yielded satisfactory results due to the complexity of the disease. Thus, developing novel treatment strategies or drugs for AD is becoming increasingly important. In long-term clinical practice, TCM formulas have been increasingly utilized for the comprehensive treatment of complex diseases, guided by a holistic view and dialectical treatment. Accumulating evidence has demonstrated that KXS, a classic formula for dementia and forgetfulness, regulates metabolism, inhibits neuroinflammation, and improves cognitive function in animal models of AD. However, the exact mechanisms through which KXS exerts its works on AD, particularly on the gut microbiota, remain unclear. In this work, APP/PS1 mice were used to systematically assess the therapeutic role of KXS in AD. In addition, multi-omics combined with experimental verification were performed to investigate the regulatory mechanism underlying KXS’s neuroprotective effects.

Significant therapeutic effects were observed with KXS including improvement of learning and memory abilities and reducing the pathological damage in the hippocampal region of APP/PS1 mice. Damage to the neuronal synapses in the hippocampus and Aβ deposition are considered key factors in the progression of AD. This study observed the ultrastructure of the synapses and Aβ deposition in neurons.

According to researches, numerous endogenous metabolites are perturbed during AD development and progression.^15^^,^^16^^,^^17^^,^^18^ Based on the UHPLC-OE/MS metabolomics platform, the endogenous metabolites in the feces were systematically analyzed after KXS administration to excavate the mechanism of KXS in the treatment of AD. In total, 43 potentially differential metabolites were screened, which were mainly involved in tryptophan metabolism, histidine metabolism, nicotinic acid, and nicotinamide metabolism, ammonia recycling, arginine biosynthesis, and amino sugar metabolism. Tryptophan metabolism, primarily through the kynurenine route, influences amyloid aggregation, excitotoxic neurotransmission, oxidative stress, uptake of neurotransmitters, and the regulation of neuroinflammation.^19^^,^^20^ Dysbiosis related to tryptophan metabolism is closely associated with AD.^20^^,^^21^ In this work, the levels of tryptophan, xanthurenic acid, and 5-methoxyindoleacetate in the APP/PS1 mice obviously decreased compared with those in the WT mice, while the levels of 5-hydroxyindole and 3-methylindole acetate were higher, which is in line with previous reports.^16^ Following KXS intervention, the levels of these tryptophan pathway metabolites were significantly reversed, showing that KXS exerts neuroprotective effects through the regulation of tryptophan metabolism. Memory loss and cognitive dysfunction are prominent symptoms of AD. Glutamic acid and urocanic acid, which are both involved in histidine metabolism, have been shown to be closely related to memory.^22^^,^^23^^,^^24^ Increasing levels of glutamic acid and urocanic acid can significantly enhance learning and memory abilities.^22^^,^^23^^,^^24^^,^^25^ Urocanic acid also facilitates short-term and long-term recognition memory acquisition, further expanding its functional role in AD.^25^ Another metabolite of histidine metabolism, 4-imidazolone-5-propanoate, is upstream of glutamate and has been shown to be involved in oxidative stress resistance.^26^ It is reasonable to speculate that 4-imidazolone-5-propanoate also influences memory and cognition. In our study, KXS significantly increased the levels of glutamic acid, urocanic acid, and 4-imidazolone-5-propanoate, thus resulting in an improvement in AD. Nicotinamide, one of the metabolites in nicotinate and nicotinamide metabolism, was reported to protect the blood–brain barrier and improve cognitive function by reducing oxidative stress and inflammation.^27^^,^^28^ In this study, nicotinamide was decreased in the APP/PS1 group and increased in KXS group, indicating that KXS could exert a neuroprotective effect via the regulation of nicotinamide metabolism. Together, the identified differential fecal metabolites mentioned above provide valuable clues for exploring the protective mechanism of KXS in AD.

There is increasing evidence supporting the crucial role of the gut microbiota in the development of AD.^29^ Additionally, disturbances in the fecal metabolites are closely related to the gut microbiota.^30^^,^^31^ Modulating the gut microbiota may be an effective approach for the treatment of AD. Polysaccharides are a key source of energy for the gut microflora.^32^ KXS contains a variety of polysaccharides, including ginseng polysaccharide, poria polysaccharide, and polysaccharide of *Polygala tenuifolia*.^33^^,^^34^ This is basically consistent with the components analysis of KXS in this work. Therefore, rebuilding of the intestinal microbial community might be a vital therapeutic mechanism of KXS in AD, and the results of this work obviously support this perspective. KXS markedly increased α-diversity and β-diversity in AD mice, indicating that KXS treatment could change the composition of the intestinal microflora of AD mice to that of the WT group. This revealed that KXS could regulate the structure of the gut microflora and restore the health status of the intestinal community. Furthermore, KXS dramatically facilitated the abundance of beneficial bacteria and inhibited the abundance of harmful bacteria. Integrating multi-omics approaches of the microbiome and fecal metabolomics provides novel insights into the microbiota–gut–brain axis, as fecal metabolites provide a link between the host and gut microbes. In the current study, the correlation analyses revealed that the phyla Deferribacteres, Bacteroidetes, Actinobacteria, and Firmicutes were correlated with the key fecal metabolites. Studies have shown decreased Firmicutes and increased Bacteroidetes in patients with AD, which were associated with augmented inflammation.^15^^,^^35^ In this work, the abundance of *Firmicutes* decreased and *Bacteroidetes* increased in APP/PS1 mice, which were reversed by intervention with KXS. Additionally, an obvious link between *Actinobacteria* and inflammation as well as neurodegenerative disease has been reported.^15^^,^^36^ Further analysis determined the gut microbes at the genus level, including *Clostridium_IV, Eubacterium, Mucispirillum, Roseburia, Blautia, Parabacteroides, Clostridium_XIVb, Prevotella,* and *Acetatifactor*. Notably, *Acetatifactor* is considered a member of a new genus and species and has been shown to be involved in colonic inflammation.^37^^,^^38^ Correlation analysis revealed a close relationship between these *Acetatifactor* and 4-hydroxybenzoic acid, succinic anhydride, glutamic acid, 4-imidazolone-5-propanoate, etc. Thus, we speculated that *Acetatifactor* influences specific receptors in the gut-brain axis by changing the levels of these fecal metabolites, subsequently participates in the pathological processes of AD. KXS may exert neuroprotective effects by regulating these gut microbes, thereby improving AD. An important component of AD pathology is inflammation of the brain. KXS could obviously inhibit inflammatory factors which exhibited great correlation with the key metabolites. These encouraging findings further verified the anti-AD mechanisms of KXS.

In the current study, we successfully utilized multi-omics combined with experimental verification to determine the protective effects of KXS in the APP/PS1 mouse model of AD. The results demonstrated that KXS effectively enhanced learning and memory in APP/PS1 mice through modulation of the gut microbiota composition. Furthermore, we identified a link between specific gut microbiota and altered metabolites, highlighting the key endogenous metabolites influenced by the gut microbiota. This study sheds new light on the role of gut microbes in the development of AD, strengthens our comprehension of the neuroprotective activity of KXS, and provides a foundation for the future development of KXS-related products.

### Limitations of the study

This work revealed that the anti-AD effect of KXS is related to regulation of metabolites influenced by gut microflora. Although the association between metabolites and neuroinflammation was identified, the underlying mechanism is still unclear. We presented that KXS can exert the anti-AD effect by regulating the composition of the gut microflora including *Clostridium_IV, Eubacterium, Mucispirillum, Roseburia, Blautia, Parabacteroides, Clostridium_XIVb, Prevotella,* and *Acetatifactor*, but their roles need to be further proved by microbiota transplantation.

## Resource availability

### Lead contact

Further information and requests for resources and reagents should be directed to and will be fulfilled by the lead contact, Zhenqiang Zhang (13333719963@126.com).

### Materials availability

This study did not generate new unique reagents.

### Data and code availability

• The 16S rRNA gene sequencing datasets generated during this study have been deposited at the National Center for Biotechnology Information (NCBI) BioProject Repository (https://www.ncbi.nlm.nih.gov/bioproject) with the project accession number PRJNA1269640 and are publicly available as of the date of publication. The 16S rRNA gene sequencing datasets generated during this study have been deposited at the National Center for Biotechnology Information (NCBI) BioProject Repository: PRJNA1269640 (https://www.ncbi.nlm.nih.gov/bioproject) and are publicly available as of the date of publication.
• Data reported in this paper will be shared by the lead contact upon request.
• This paper does not report original code.
• Any additional information required to reanalyze the data reported in this paper is available from the lead contact upon request.

## Acknowledgments

This research was funded by grants from the National Natural Science Foundation of China (nos. 82305087 and 82274612); Postdoctoral Research Foundation of China (no. 2022M711080); Joint Research Fund of Science and Technology R&D Plan of Henan Province (no. 222301420068); Scientific and Technological Project of Henan Province (no. 242102311258); Program for Science & Technology Innovation Talents in Universities of Henan Province (nos. 25HASTIT062 and 23HASTIT044). Key research and development project of Henan Province (no. 231111312900); Key research projects of university of Henan Province (no. 23A310013).

We would like to thank large instruments and equipment sharing platform (Academy of Chinese Medical Sciences, Henan University of Chinese Medicine) for help with mass spectrometry.

## Author contributions

Conceptualization, H.F.M. and Z.Q.Z.; methodology, P.W.; Investigation, Z.Y.Y., Q.Q., and W.P.W.; writing – original draft, H.F.M. and Z.Y.Y.; writing – review and editing, Z.S.X., Y.R.S., and Z.H.L.; funding acquisition, H.F.M., Z.Q.Z., Y.R.S., and Z.H.L.; resources, Z.S.X., J.Y.S., and Z.Q.Z.; supervision, P.W.; data curation, H.F.M., X.W.Z., and Y.F.S.

## Declaration of interests

The authors declare no conflict of interest.

## STAR★Methods

### Key resources table

**Table:** 

REAGENT or RESOURCE	SOURCE	IDENTIFIER
**Antibodies**		

Aβ_1-42_ anti-rabbit monoclonal antibody	Abcam	Cat. No.ab201060; RRID: AB_2818982

**Chemicals, peptides, and recombinant proteins**		

HPLC-grade Methanol	Fisher Scientific	Cat. No. A452-4
HPLC-grade Acetonitrile	Fisher Scientific	Cat. No. A998-4
LC/MS-grade Formic acid	Fisher Scientific	Cat. No. A117-50

**Critical commercial assays**		

TNF-α kit	Elabscience Biotechnology Co., Ltd	Cat.no. EL-M3036
Il-6 kit	Elabscience Biotechnology Co., Ltd	Cat.no. EL-M0044
Il-1β kit	Elabscience Biotechnology Co., Ltd	Cat.no. EL-M0037

**Deposited data**		

16s rRNA sequencing data	Mouse stool	NCBI Bio project accession number PRJNA1007862 https://www.ncbi.nlm.nih.gov/bioproject

**Experimental models: Organisms/strains**		

APP/PS1 mouse	Beijing huafukang biotechnology Co., Ltd	SCXK(Jing)2019-0008
C57BL/6J mouse	Beijing huafukang biotechnology Co., Ltd	SCXK(Jing)2019-0008

**Software and algorithms**		

Compound Discoverer 3.1	Thermo Fisher Scientific	N/A

**Other**		

*Ginseng Radix*	Henan Zhang Zhongjing Da Pharmacy Co., Ltd	N/A
*Polygalae Radix*	Henan Zhang Zhongjing Da Pharmacy Co., Ltd	N/A
*Acori Tatarinowii Rhizoma*	Henan Zhang Zhongjing Da Pharmacy Co., Ltd	N/A
*Poria*	Henan Zhang Zhongjing Da Pharmacy Co., Ltd	N/A

### Experimental model and study participant details

#### Animals and drug administration

In this study, APP/PS1 mice were used as experimental animals. 48 male APP/PS1 mice and 12 littermate wild-type (WT) male C57BL/6J mice were obtained from Beijing huafukang biotechnology co., Ltd. The animals were raised in SPF animal experimental center of Henan University of Traditional Chinese medicine (ethical lot number: DWLLGZR202202145). Tests were conducted on mice that were approximately 6 months old and weighed between 25 and 33 g. The animals were reared in separate cages with 6 animals in each cage, fed with standard rodent feed and water. In advance of the experiments, all mice were acclimated to a chow-and-water environment for 7 days. The cycle of light/dark was 12 h, at temperature 22°C–25°C, humidity 40%–70%. All experiments were conducted in accordance with Chinese legislation regarding experimental animals. The APP/PS1 mice were randomly divided into the following 5 groups (*n* = 12 per group): model group (APP/PS1), KXS group (Low 0.7 g/kg/d, Middle 1.4 g/kg/d, High 2.8 g/kg/d) and Donepezil group (2 mg/kg/d).^39^ C57BL/6J mice served as the control group. A 300 mL dose of KXS was administered orally once daily for 8 weeks to mice in the KXS group, while the APP/PS1 group and control group received purified water orally.

### Method details

#### The chemical components analysis of KXS

To obtain effective ingredients of KXS, four component herbs, were soaked in distilled water (solvent:sample = 5:1, v/w) for 1 h. Then boiled it twice, each time for 45 min. The extracted solutions were combined, filtrated and concentrated (enriched to 0.2 g/mL). To assess the quality of water extracts, an Ultimate 3000 ultra-high performance liquid chromatography (UHPLC) system coupled to a Orbitrap Exploris (OE) 240 mass spectrometer (Thermo Fisher Scientific, Bremen, Germany) was employed. Meanwhile, KXS was injected into the UHPLC-OE/MS system to examine the results of the principal components. The column temperature was 35°C and the injection volume was 2 μL. The mobile phase was composed of 0.1% aqueous acetic acid (A) and methanol (B) at a flow rate of 0.2 mL/min. Chromatographic conditions are as follows: 0–4 min, 5%–20% B; 4–9 min, 20%–30% B; 9–17 min, 45%–55% B; 17–18 min, 55%–60% B; 18–23 min, 60%–75% B; 23–27 min, 75%–95% B; 27–30 min, 95% B; 30–30.1 min, 95%–5% B; 30.1–35 min, 5% B. Mass spectrum conditions are as follows: Sheath gas and auxiliary gas flow rates were 35 and 12 arbitrary unit, respectively. Capillary voltage was 3500 V (positive) and 3000 V (negative), respectively. The ion transfer tube and vaporizer temperature were separately 350°C and 325°C. HCD collision energies (%) was 20%, 40% and 60%, and scan range (m/z) was 200–2000.

#### Morris water maze test (MWM)

MWM platform was divided into four quadrants according to the orientation, and the hidden platform was fixed in the second quadrant, which was 1–2 cm below the water surface. During the test, mice were put into the water surface from the midpoint of the edges of the four quadrants, and the time required for mice to find the platform was recorded. If the mice did not find the platform within one minute, guide the mice to the platform and stay for about 20 s for 5 days. On the sixth day, the hidden platform was removed, and the mice were put down from the four quadrants in turn. The number of times each mouse crossed the platform within 60 s and the time it stayed in the second quadrant was recorded.

#### Y-maze test

The Y maze includes three equal-length arms at 120° to each other and a connecting area, which was used for detecting spatial working memory. Before the test, the mice were placed in the behavioral testing room for 1 h to make them to acclimate to the surroundings. Subsequently, the mice were placed into the maze at a specific location and permitted to freely explore the arms in a short time. The number of arm entries and alternations was then recorded to calculate the percentage of alternation behavior.

#### Nest-building test

Cognitive behavioral function also could be assessed with nest-building test. Before the experiment, the mice were placed in separate cages and filled with new padding. Then, same volume of compressed cotton was added the in the cage at 6 p.m. After 12 h, the nesting behavior of mice were evaluated according to the utilization of absorbent cotton.

#### Transmission electron microscope (TEM)

The brain tissue of mice was taken and fixed in 2.5% glutaraldehyde fixed solution, and kept in the refrigerator at 4°C overnight. After rinsing, 1% osmium acid was fixed for 1.5 h. Gradient propanol and embedding solution were separately used for dehydration and embedding. Slice with ultrathin microtome and dye with uranium acetate-lead citrate. The images were observed and taken through TEM.

#### Immunofluorescence staining

Brain tissues were made into frozen sections with a thickness of 10 μm. The frozen sections of brains were incubated with primary and secondary antibodies. Then DAPI was used to stain the nucleus. A microscope was used to observe and take pictures.

#### 16S rRNA microbial community analysis

30 ng qualified genomic DNA samples were added into PCR reaction system with corresponding fusion primers. Then PCR reaction parameters were set for PCR amplification. Agencourt AMPure XP magnetic beads were used for purifying PCR amplification products. Elution Buffer was applied for dissolving the samples. Finally, the database was constructed after labeling them. Detection of the fragment range and concentration of the library was performed on Agilent 2100 Bioanalyzer. According to the size of inserted fragments, the qualified libraries were sequenced on the platform of HiSeq. The QIIME2.0 software package was used for analyzing the original reads, which was obtained from the sequencing of fecal samples, including filtering, denoising and merging, in order to ensure the accuracy of operational Taxonomic Units (OTUs) clustering and subsequent analysis. The α diversity and the linear discriminant analysis combined with effect size (LEfSe) were separately applied for assessing diversity and similarity among individual microbial groups, and dominant microbial group in each group.

#### Fecal metabolomic analysis

Took about 100 mg of fecal samples, added 10 times the volume of precooled 50% methanol (add 1 mL of 50% methanol to 100 mg of samples), homogenize, centrifuge at 3,500 rpm, and took the supernatant. Added 2 times of methanol containing internal standard compound, vortex mixing, centrifuged at 13,000 rpm, took supernatant, and dried the sample with vacuum centrifugal concentrator. Added 200 μL methanol for redissolution, centrifuged twice at 13,000 rpm, collected the supernatant in a sample injection vial. 40 μL was collected from every samples and mixed as quality control (QC) samples. All the samples and QCs were detected and analyzed with Thermo scientific UHPLC-OE/MS system under positive and negative ion modes, with automatic sample injection at 4°C throughout the process. The LC-MS data of fecal samples were processed by Compound discoverer 3.1, and the metabolites were identified. For a more detailed analysis, pattern recognition methods were employed to identify metabolic differences between groups, such as principle component analysis (PCA). To identify the differences between the AD and WT groups, as well as the KXS group, orthogonal partial least squares (OPLS)-discriminant analysis (DA) plots and S-plots were created, respectively. Furthermore, the KXS group and APP/PS1 group were analyzed using the T-test. According to fold change (FC) values > 1 and *p* < 0.05, the differences of all ions detected in the fecal samples of the KXS and APP/PS1 group were analyzed. Exploring related metabolic pathways using KEGG database.

#### Enzyme-linked immunosorbent assay (ELISA)

Brains in different groups were collected and processed as described above. The levels of inflammatory factors were detected with ELISA kits according to the corresponding manufacturer’s instructions (Nanjing Jiancheng Bioengineering Institute, Nanjing, China).

### Quantification and statistical analysis

GraphPad Prism 8.0 software was applied for statistical analysis. One-way ANOVA was performed on the experimental data. Data were expressed as Mean ± SEM, and *p* < 0.05 was considered significant.
